# Preference-oriented quality of life monitoring and linkage with clinical registry data: study protocol of a randomised clinical trial in patients with lung cancer (LePaLuMo Study)

**DOI:** 10.1186/s13063-025-09102-3

**Published:** 2025-09-16

**Authors:** Patricia Lindberg-Scharf, Martin Emmert, Michael Koller, Florian Gürtler, Brunhilde Steinger, Jacqueline Müller-Nordhorn, Florian Zeman, Sophie Friebel, Ksenia Ibler, Jan Kurz, Thomas Stangl, Monika Klinkhammer-Schalke, Vinzenz Völkel

**Affiliations:** 1 Tumor Center Regensburg, Center of Quality Management and Health Services Research, University of Regensburg, Am BioPark 9, 93053 Regensburg, Germany; 2 Institute for Healthcare Management and Health Sciences, Faculty of Law, Business and Economics, University of Bayreuth, Universitätsstraße 30, 95447 Bayreuth, Germany; 3 Bavarian Cancer Registry, Bavarian Health and Food Safety Authority, Schweinauer Hauptstraße 80, 90441 Nuremberg, Germany; 4 Center for Clinical Studies, University Hospital Regensburg, Franz-Josef-Strauß-Allee 11, 93053 Regensburg, Germany

**Keywords:** Quality of life, Lung cancer, Definitive RCT, Patient empowerment, Quality of life monitoring, Patient and physician preferences

## Abstract

**Background:**

In routine oncological care, the implementation of disease-related quality of life (QoL) is still an open matter. In a complex intervention, a QoL monitoring system including tailored therapeutic options has been designed, implemented, and its effectiveness has been demonstrated in two randomised trials in patients with breast and colorectal cancer. The next step is to extend the usability of the QoL monitoring system for patients with other cancer diagnoses and in other regions. Necessary adaptations include an electronic measurement of QoL and consideration of patient and physician preferences. The present randomised trial investigates the effectiveness of this adapted QoL monitoring system in patients with lung cancer in two regions in Bavaria, Germany.

**Methods:**

In this 2-arm randomised, prospective, pragmatic, multicentre clinical trial with one intervention and one control group, QoL of primary lung cancer patients is assessed with an electronic patient- and physician-oriented QoL monitoring system using the EORTC QLQ-C30 and QLQ-LC29 questionnaires at study entry and at 1, 2, 3, 4, 5, and 6 months during follow-up care. The QoL data of each patient are linked with clinical data from the Bavarian Cancer Registry for the purpose of data analysis. In the intervention group, the results of QoL monitoring are automatically transferred to a QoL profile including 8 dimensions on scales of 0–100 (cut-off “need for QoL therapy” < 50 points). QoL results are obtained in real-time by patients and their treating physicians. To treat QoL deficits, a multi-professional network of healthcare providers is established. In the control group, QoL is also measured, but neither patients nor treating physicians have access to the results. The investigators expect that the proportion of patients in both groups with a need for QoL therapy (< 50 points in at least one dimension of the QoL profile) will be lower in the intervention group than in the control group at the primary endpoint 6 months after study entry.

**Discussion:**

This is the first study investigating the effectiveness of a QoL monitoring system based on patient- and physician-oriented preferences with a high degree of generalisability by including inpatient and outpatient care as well as different study regions.

**Trial registration:**

ClinicalTrials.gov NCT06252233. Registered on February 2024.

## Background

### Background and rationale

Lung cancer is the leading cause of cancer mortality, accounting for approximately 1.80 million deaths worldwide in 2020 [1]. In Germany, in 2019/2020, lung cancer was the most common cause of cancer death in men (22%) and the second most common cause of cancer death in women (16%). In terms of the number of new cases, lung cancer was the second most common tumour location, with 35,890 new cases in German men, and the third most common tumour location in women, with 23,720 new cases. While the incidence rates for men have been declining since the 1990 s, they are increasing for women. In the majority of cases, patients are being diagnosed at an advanced stage [2].

The quality of life (QoL) of lung cancer patients is considerably impaired. Zabora et al. (2001) reported that the level of distress is highest in this patient group than in patients with 13 other common tumour diseases [3]. Over 90% of patients with advanced lung cancer suffer from fatigue, loss of appetite, shortness of breath, cough, and pain [4]. A systematic review investigating the QoL of patients with small cell lung cancer (SCLC) revealed a negatively impaired QoL in most domains compared to that of the general population with physical functioning being most affected [5]. In patients diagnosed with non-small cell lung cancer (NSCLC), Poghosyan et al. (2013) identified pain, dyspnea, coughing, and fatigue as the most common symptoms in the first few months after surgical resection [6]. Such unmet supportive care needs (e.g., physical, emotional, social, psychological, informational, spiritual, and practical needs) are associated with poorer QoL in patients with lung cancer [7].

Therefore, maintaining or improving QoL is an important therapeutic goal in the treatment of patients with lung cancer. Studies investigating the effectiveness of monitoring systems for QoL or adverse events in cancer patients have shown positive effects on QoL [8, 9] and survival [10–12] in routine care. A recent study involving not only health professionals but also patients with colorectal, breast, or gynaecological cancers in their QoL monitoring demonstrated positive effects on physical functioning and self-efficacy during chemotherapy [13]. Thus, QoL monitoring can have positive effects on patient empowerment which can be defined “as the increasing ability of patients to actively understand, participate in, and influence their health status” [14] (p. 1). Accordingly, a systematic review detected positive effects of web-based interventions on patient empowerment compared with usual care [15].

Within our research group, a clinical pathway to improve the QoL of cancer patients has been developed, implemented, and evaluated in a complex intervention [16], including theory development [17], modelling (phase I) [18], and exploratory study (phase II) [19]. Two randomised trials (phase III) showed a significantly better QoL of patients with breast cancer [9] and colorectal cancer [8] receiving systematic QoL diagnosis and therapy during follow-up care. A unique feature of this intervention is that QoL monitoring is embedded in inpatient as well as outpatient care.

The next step is to extend the usability of the QoL pathway so that it can be used by patients with other cancer diagnoses and in other regions (phase IV). Therefore, in the present study, QoL will no longer be measured on a paper-based basis but rather by using an electronic patient- and physician-oriented QoL monitoring system based on previous work [20]. An additional special feature of the intervention is that, in addition to their physicians, patients will also be actively involved in their QoL monitoring and can view the results. The present randomised trial investigates the effectiveness of this adapted QoL monitoring system in patients with lung cancer in two regions of Bavaria, Germany. Additionally, QoL data will be linked with clinical cancer registry data to investigate the feasibility of using the combined dataset to answer relevant medical and therapeutic questions.

### Objectives

The primary objectives are as follows:

1. Assessing the effect of a patient- and physician-oriented QoL monitoring system on the improvement of QoL in lung cancer patients
2. identification of benefits and barriers in linking QoL data with cancer registry data

The secondary objectives are as follows:

(3) improving the self-efficacy of patients in dealing with lung cancer by actively integrating patients into their QoL monitoring and sensitising them to the various dimensions of their QoL
(4) improvement of patient-physician communication
(5) evaluation of the acceptance of the QoL monitoring system from the perspective of patients and physicians
(6) identification of differences in the use and evaluation of the QoL monitoring system in specific subgroups (e.g., age, sex)

## Methods

### Trial design

The study is a two-armed, randomised, controlled, prospective, pragmatic, multicentre clinical trial with an intervention group and a control group. QoL of primary lung cancer patients will be assessed with an electronic patient- and physician-oriented QoL monitoring system at study entry and after 1, 2, 3, 4, 5, and 6 months of follow-up care. The subjects are randomly assigned (1:1 allocation ratio) to (1) an intervention group with tailored QoL diagnosis and therapy or (2) a control group with routine follow-up care. QoL data of each patient will be linked with clinical data from the Bavarian Cancer Registry for the purpose of data analysis. Thus, it is not necessary to collect patient-, tumour-, or treatment data exclusively for the purpose of this study, but rather existing data which already has been officially registered can be used. This saves a considerable amount of temporal and financial effort. This protocol is compliant with the SPIRIT 2013 reporting guidelines [21].

### Study setting and eligibility criteria

Eight hospitals treating patients with lung cancer in Bavaria, Germany participate in patient recruitment. To achieve adequate participant enrolment there is regular contact between the study unit and the clinicians who are responsible for patient recruitment in the eight hospitals.

The following inclusion criteria apply: (1) primary diagnosis of lung cancer (C33/C34); (2) treatment at one of eight recruiting centres (University Hospital Regensburg, Hospital Barmherzige Brüder Regensburg, Hospital St. Maria Donaustauf, Hospital St. Elisabeth Straubing, Hospital Bayreuth, Sana Hospital Coburg, Hospital Kulmbach, Hospital Bamberg); (3) a time interval between the date of histology and the date of study entry not exceeding 6 months; (4) informed consent (a model consent form can be made available on request).

The exclusion criteria are as follows: (1) unavailability of a study clinician for patient recruitment (e.g., illness); (2) misclassification of the patient in the candidate list (e.g., no primary diagnosis, no lung tumour); (3) refusal of trial participation by the coordinating practitioner; (4) living outside the defined study region (Germany, Bavaria: Upper Palatinate, Lower Bavaria, Upper Franconia); (5) age under 18 years; (6) pregnancy/breastfeeding; (7) inability to fill out the QoL questionnaire (physical, psychological, cognitive, language reasons); (8) refusal of trial participation.

**Fig. 1:**
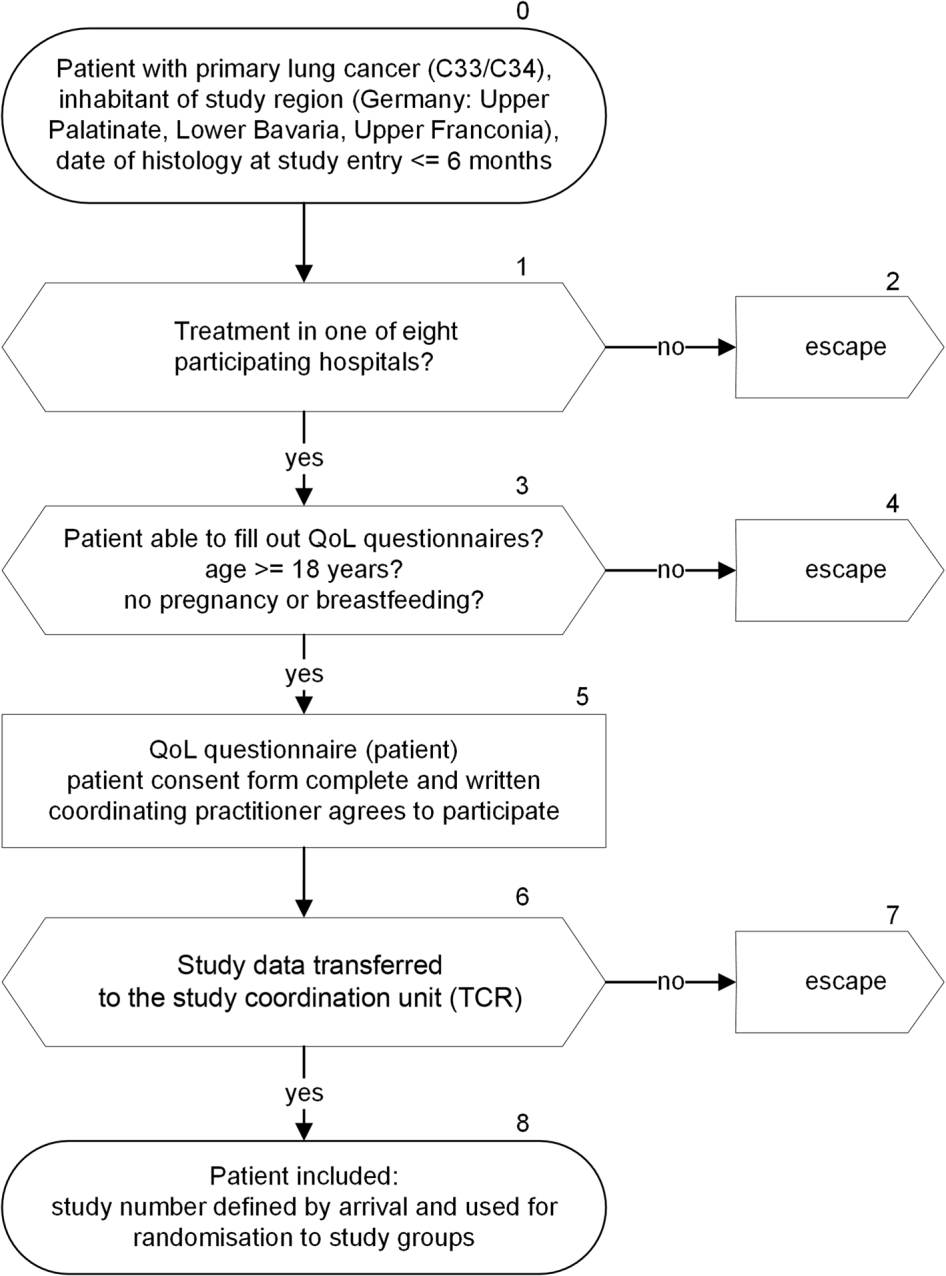
Selection of patients

To ensure high external validity [22], there will be no exclusion of elderly patients or according to tumour stage or patient sex. Furthermore, no constraints will be made according to the type of therapy. However, these factors will be accounted for in the data analyses. External validity is also maintained by population-based inclusion of patients from urban and rural areas and by implementing the QoL monitoring system in routine follow-up care. The selection of study participants is shown in Fig. 1.

During follow-up care patients are treated by their coordinating practitioners (CPs), who are defined as those physicians who care for the lung cancer patients after hospital stay in the outpatient setting (oncologists, pneumologists, internists, general practitioners).

### Randomisation and blindness

Subjects who meet inclusion criteria but none of the exclusion criteria are randomly assigned to the intervention or control group (1:1 allocation ratio) with the underlying parameters block randomisation and stratified randomisation with the strata gender (female/male) and centre. Random lists were generated by the Center for Clinical Studies (CCS) using the software SAS 9.4. Based on these random lists of digits, a coworker of the Tumor Center Regensburg (TCR), otherwise not involved in the trial, creates paper cards for each of the six recruiting centres specifying the study group for male and female participants and inserts them in serially numbered opaque, sealed envelopes. This stack of envelopes is kept in a locked safe at the study coordination unit of TCR, which is separated from the study research unit of UR/TCR in terms of space, organization and personnel to which only the two study coordinators have access. This procedure ensures that study coordinators who are responsible for randomisation of study patients are kept blind to the randomisation sequence. The eligibility of the study patients is determined by study clinicians in each of the six recruiting centres. If all criteria are fulfilled and the patient has given informed consent the study clinician sends the recruitment document to the study coordination unit (TCR) by fax. The exact date and time of fax entry determine the order in which patients from each centre are randomised.

The recruiting clinicians who determine study eligibility are blinded to the randomisation sequence. CPs and patients cannot be blinded to allocation for practical reasons because the intervention requires that intervention group patients and their CPs have access to the results of QoL monitoring. However, the study staff conducting the patient and physician evaluations after 3 and 6 months will be blinded to the condition of allocation.

### Patient- and physician-oriented QoL monitoring system

In order to provide a comprehensible and manageable QoL monitoring system for both patients and physicians, we aim to focus on what matters most to both patients and physicians, i.e., to concentrate on the most relevant QoL dimensions for both study groups, respectively. Therefore, prior to the initiation of the RCT we developed and performed two separate discrete choice experiments (DCEs) to elicit and compare patients’ and physicians’ preferences for relevant QoL dimensions during lung cancer treatment. DCEs are increasingly used in the healthcare context to inform on preferences for healthcare services such as treatment choices [23]. DCEs are a stated preference method in which (survey) data are used to elicit the preferences of patients, physicians, and others based on a series of hypothetical choice scenarios [24].

Therefore, our study used a mixed methods approach. First, we performed two systematic search procedures on Medline (via PubMed) and the Cochrane Library to identify literature that aimed to identify and evaluate the importance of QoL dimensions during lung cancer treatment from the perspective of both patients and physicians. Second, we qualitatively surveyed 16 randomly selected lung cancer patients and 16 physicians to learn more about the importance of the identified QoL dimensions during lung cancer treatment. The respondents were sent a short survey via postal email before conducting semi-structured interviews regarding the importance of the identified QoL dimensions. The aim of the semi-structured interviews was to explore in greater depth the stated preferences, to determine the most relevant QoL dimensions as well as corresponding levels, to clarify the wording, and to evaluate the comprehensibility of hypothetical choice tasks for the DCE.

Third, we developed one online-based survey version of the DCE for each study group. Both survey instruments were similar and consisted of four parts. First, respondents were presented with relevant QoL dimensions (e.g., pain, shortness of breath, emotional impairment) as well as short descriptions and were asked to rate each QoL dimension on a 1–5 scale (1 = not all important; 5 = very important). Furthermore, we asked both patients and physicians to select the single most important QoL dimension during lung cancer treatment. Second, all respondents responded to the DCE survey block. Here, both DCE experiments were designed using Sawtooth Software Lighthouse Studio 9.14.2. Thereby, DCEs perform pairwise comparisons of hypothetical alternatives (i.e., differently configured health statuses during lung cancer treatment) and ask respondents to choose between them. We used full profile designs (i.e., each choice set included all six QoL dimensions) and the balanced overlap method to achieve standard errors below 0.05 for main effect utilities and 0.10 or smaller for interaction effects and the highest D-efficiency score [25]. We designed the choice sets in both surveys as forced-choice tasks (i.e., respondents had to choose one of two hypothetical health status by making trade-off decisions). As stated above, health statuses differed in six attributes with three levels each following current DCE guidelines [26]. Third, we evaluated both the clarity and comprehensibility of different QoL monitoring system designs. Therefore, we presented different ways of presenting QoL development over time based on international practices. Fourth, we asked for general sociodemographic information (e.g., age, gender) as well as treatment-related information. The questionnaire was pilot tested for clarity and understanding with 8 lung cancer patients and 9 physicians and was slightly modified accordingly. Both surveys were conducted online, i.e., patients and physicians were contacted via email or received a paper-based flyer that contained information about the survey and a link to participate online (web-based survey). Based on the results of both DCE experiments, we selected the eight most relevant QoL dimensions for inclusion in our QoL monitoring system and listed each QoL dimension depending on its importance.

#### Sample size for DCEs

Johnson’s rule-of-thumb would lead to a sample of at least 75 participants regarding our DCE specifications (i.e., ten choice tasks per respondent, two alternatives, three levels per attribute as maximum) [27]. However, we aimed to double this number (i.e., at least 150 participants) following more advanced recommendations for statistical robustness [28, 29].

### Procedure

**Fig. 2:**
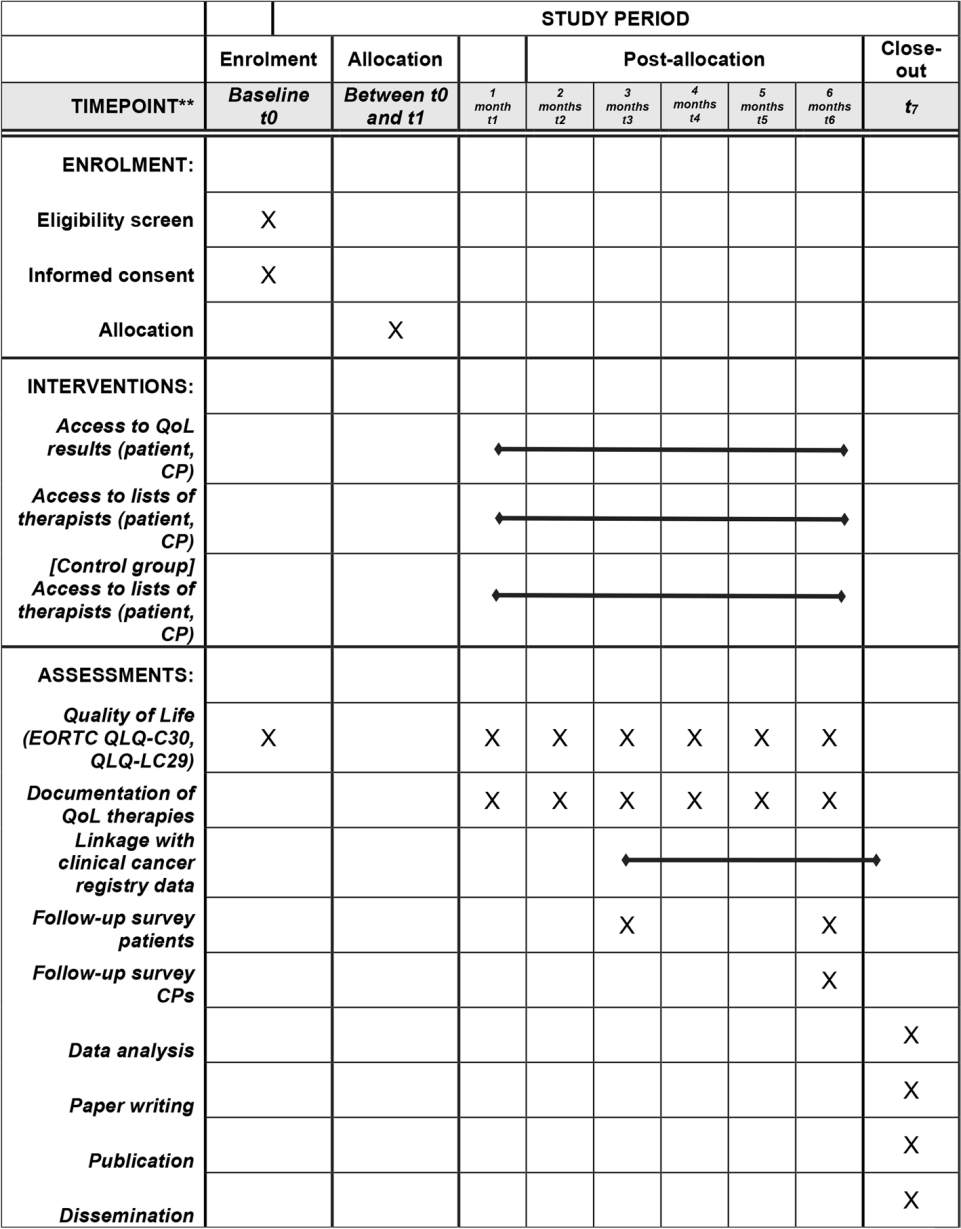
Schedule (SPIRIT diagram) of enrolment, interventions, and assessments

All participants (in the intervention and control groups) complete two QoL questionnaires (European Organisation for Research and Treatment of Cancer (EORTC) QLQ-C30 and QLQ-LC29 [30–32]) at the following time points: baseline (during the first 2 months of confirmed diagnosis of primary lung cancer) and 1, 2, 3, 4, 5, and 6 months after baseline (see. Figure 2). The first QoL measurement (baseline) takes place in the hospital by using a paper–pencil version of the QoL questionnaires for practical reasons. QoL data are entered manually into the online QoL monitoring system by a study coordinator at the study coordination unit so that patients and their CPs have online access to the results of the baseline QoL monitoring. All further QoL measures (1, 2, 3, 4, 5, and 6 months) will be completed on the patient`s mobile phone or computer via a secure web portal. For this purpose, after study entry each patient receives an email from the study coordination unit at the Tumor Center Regensburg with a link to activate the user account and assign a password. At 1, 2, 3, 4, 5, and 6 months after baseline each patient receives an email with a link to the QoL measurement, which is valid for two weeks. Patients who have not completed their QoL monitoring will receive two email reminders (after seven days and after 12 days). Once a QoL measurement has been completed it can no longer be edited. If participants have missed two QoL measurements in a row or if the QoL measurement at 6 months (primary endpoint) is missing they will be contacted by phone by one of the two study coordinators of the study coordination unit to check if there are any difficulties (e.g., use of the web application, internet access). If a participant has no regular access to the internet, there is also the possibility of a paper-based QoL measurement. For this purpose, QoL questionnaires are sent to the patient`s home by post at each timepoint for QoL measurement including a stamped return envelope.

A user account is also created for each CP providing follow-up care of the study patients. Each CP has access to those study patients who have been assigned to his/her own practice.

Patients will be asked at 3 and 6 months after baseline and CPs at 6 months after baseline to complete an online questionnaire to evaluate their acceptance of the QoL monitoring system. All patients are treated according to the follow-up regimen of the German S3-guideline for lung cancer [33].

#### Intervention group

**Fig. 3:**
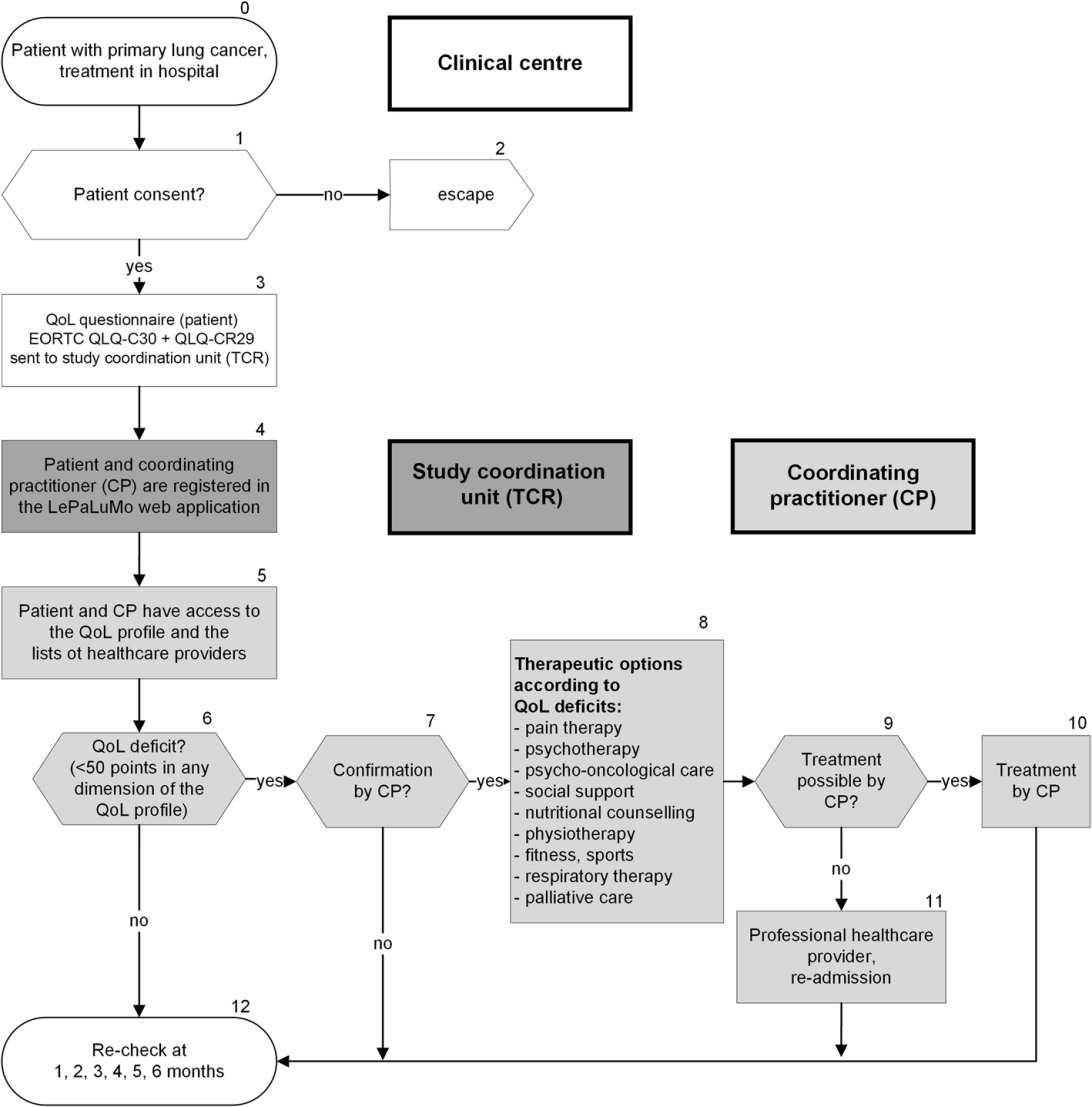
Patients in the intervention group (quality of life (QoL) pathway) following recruitment for the randomised trial. This pathway is considered for routine clinical practice

Patients in the intervention group receive, in addition to routine follow-up care [33], tailored QoL diagnosis and therapy for 6 months: After each QoL measurement, patients and their CPs have direct access to the results in the form of a QoL profile via a web application. In the case of an exclusively paper-based QoL measurement, patients receive their QoL profiles by post. The profile shows QoL on scales of 0–100 (worst to optimal QoL) comprising global QoL and seven different dimensions considering somatic (i.e., pain), psychological (i.e., emotional functioning), and social aspects (i.e., family life) (EORTC QLQ-C30 [30]). The QoL profile also takes into account specific discomfort that lung cancer patients suffer from (e.g., shortness of breath) (EORTC QLQ-LC29 [32]) (see Fig. 3). These dimensions were selected based on patient- and physician preferences that were determined in a preliminary study with DCEs as described above as well as on the results of two previous RCTs in breast cancer [6] and colorectal cancer patients [7]. A cut-off score < 50 points indicates a need for QoL therapy in each QoL dimension [6–9].

CPs are regularly informed about their patients' QoL by automatically receiving information via email. They have access to their patients` QoL profiles as well as lists with professional healthcare providers so that specific complaints can be clarified at the next doctor-patient consultation and tailored therapies to improve QoL can be initiated.

For this purpose, the following therapeutic options for QoL improvement in patients with lung cancer have been defined based on previous studies of the complex intervention [6–8] and the expertise of the participating study clinicians:

- Pain therapy
- Psychotherapy
- Psycho-oncological care
- Social support
- Nutritional counselling
- Physiotherapy
- Fitness and sports
- Respiratory therapy
- Palliative care

To provide continuous medical education, professional healthcare providers in this multi-professional network must meet in quality circles. As a result of the previous two RCTs (6; 7), regional network structures for QoL therapies have been established and are still available in the study region Upper Palatinate/Lower Bavaria. The Upper Franconia was added as a new study region to enhance the generalisability of the study results by implementing QoL monitoring in another region. CPs and patients obtain complete lists of the network of healthcare providers practicing in their region via the web portal or, if desired, by post to contact them easily for QoL therapy.

No recommendations are made regarding specific therapeutic options. The QoL profile provides a descriptive aid, but the decision of whether to initiate any specific QoL therapy is made by the CP and the patient. The QoL pathway of the patients in the intervention group is shown in Fig. 3.

#### Control group

In the control arm, QoL of patients is also assessed with the same QoL monitoring system used in the intervention group at study entry and at 1, 2, 3, 4, 5, and 6 months, but neither patients nor CPs have access to the results of their QoL measurements (no access to QoL profiles). However, complete lists of the network of local healthcare providers are also available for control group patients and their CPs via the web portal or if desired by post. Patients receive standard follow-up care according to the S3-guideline for lung cancer [33].

### Measures

#### Quality of life (patient)

**Fig. 4:**
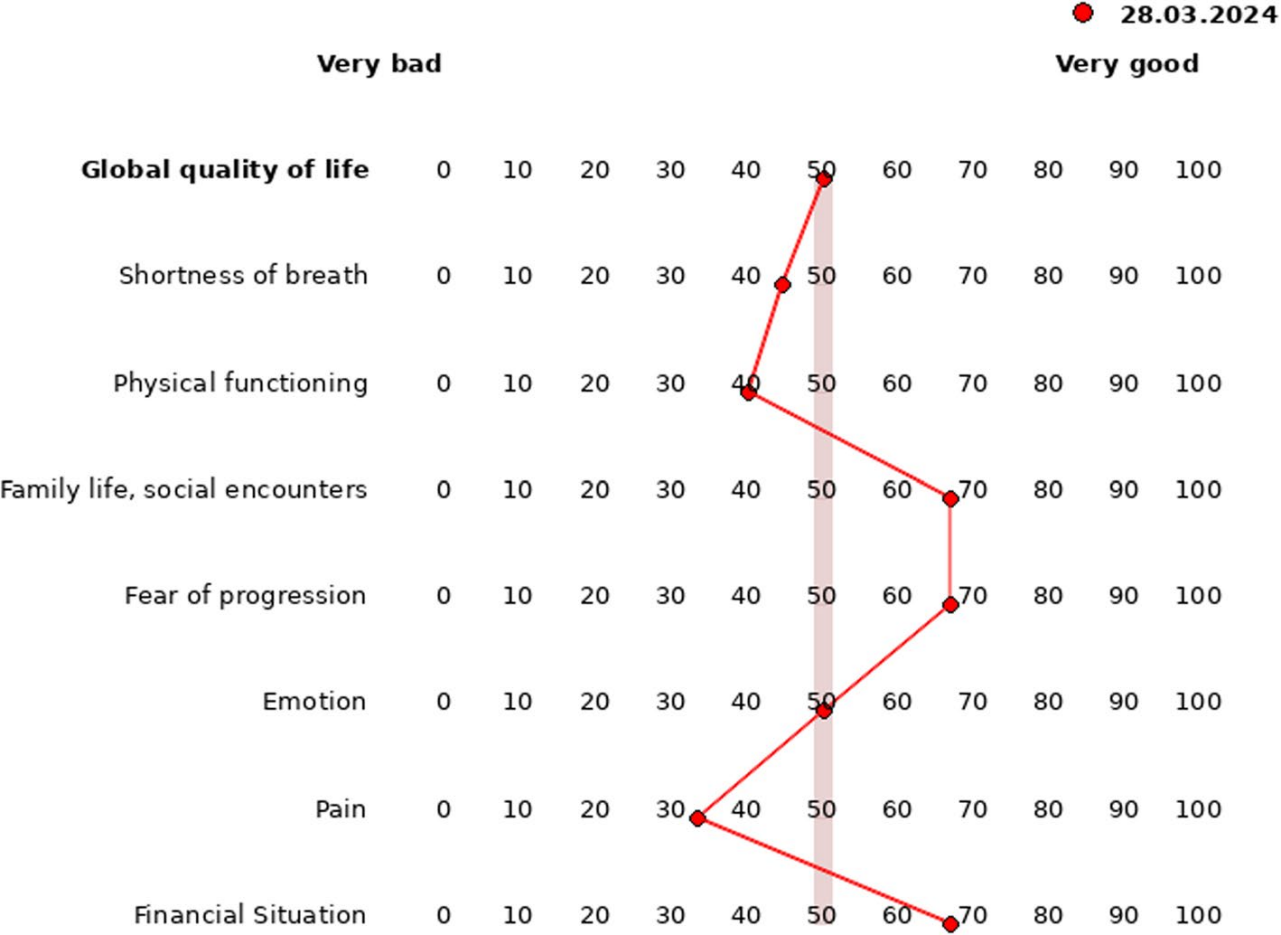
QoL profile of patients with lung cancer in the intervention group. The cutoff for a need for QoL therapy is < 50 points

QoL is measured by using the validated QoL questionnaires of the European Organisation for Research and Treatment of Cancer (EORTC), consisting of a core module (EORTC QLQ-C30 [30]) and a module for lung cancer patients (EORTC QLQ-LC29 [32]). The questionnaire is a self-administered instrument with a multidimensional structure measuring QoL on a four-point Likert scale or rather on a seven-point Likert scale for the global QoL dimension. Satisfactory internal consistency, good retest-reliability, and good construct and clinical validity have been demonstrated [30, 32]. For every dimension scores are uniformly transferred to a scale from 0 (= very bad QoL) to 100 (= very good QoL). A cut-off score < 50 points indicates a need for QoL therapy in each QoL dimension [6–9]. This decision criterion was chosen because the present study aims to highlight the patient’s perspective of subjective impairment. This is operationalised by dichotomising symptom scores with a majority of “quite a bit” and “very much” responses to the “bad” side of the spectrum (< 50) and “not at all” and “a little” responses to the “good” side (≥ 50). For outcome measurement, eight scales have been selected according to the results of both previous RCTs (9; 8) and our preliminary study examining patient and physician preferences: global QoL, shortness of breath, physical functioning, social functioning, fear of progression, emotional functioning, pain, and financial situation (Fig. 4).

#### Clinical cancer registry data

Based on the informed consent of the study patients and an approval from the Scientific Advisory Board of the Bavarian Cancer Registry, clinical cancer registry data from the Bavarian Cancer Registry will be linked with the QoL data (EORTC) of the study patients. Therefore, relevant study data are obtained directly from the registry preventing the collection of duplicate data (e.g. diagnostic information, therapy procedures). Details of the data linkage are described in the “ Data management and privacy” section.

#### QoL therapies

After 3 and 6 months, patients provide information about the QoL therapies they have used during the last 3 months via the web portal or, in the case of exclusively paper-based QoL measurements, per post.

#### Follow-up survey (evaluation of the QoL monitoring system)

1. Use of the QoL monitoring system [34]:
  Patients will be asked via the web portal or post at 3 and 6 months after baseline to answer questions relating to comprehensibility, user-friendliness, relevance, usefulness, patient-physician communication, and self-efficacy.
  CPs will be asked via the web portal at 6 months after baseline to answer questions related to patient-physician communication, future intention to use the QoL monitoring system and willingness to recommend it, and effects on quality of care.
2. Acceptance of the QoL monitoring system [34]:
  For this purpose, an overview of possible acceptance models (e.g., the Unified Theory of Acceptance and Use of Technology (UTAUT) [35, 36]) is given by means of literature research and their applicability in our RCT is evaluated.
  Patients will be surveyed via the web portal or post 3 months after baseline.
  CPs will be surveyed via the web portal 6 months after baseline.

### Ethics and monitoring

This study received full ethical approval from the ethics committee of the University of Regensburg (internal reference number 23-3303_1-101). The trial is registered with ClinicalTrials.gov, trial registration number NCT06252233.

All adult patients with a new primary diagnosis of lung cancer can be included in the study (see exclusion criteria). The completion of QoL questionnaires (approximately 15 min) could be time-consuming for patients. It must also be considered that patient empowerment, as investigated in the intervention arm (patients have access to their QoL profiles), represents a risk for intervention group patients. However, an international RCT did not provide any evidence for the potential harm of patient empowerment by using a monitoring system [13]. Moreover, all study patients are under the continuous care of a CP who can react in the unlikely case of potential harm. Additionally, the CPs of the patients in the intervention group receive results of QoL measurements so that they can support them in cases of impaired QoL. The expected benefit is an improved detection of dimensions with reduced QoL in the intervention arm. It can also be assumed that control group patients will benefit from participating in the study, as their CPs will be sensitised to supportive, patient-oriented therapeutic options by having access to address lists of the multi-professional care network. The previously conducted RCTs on breast cancer patients [9] and colorectal cancer patients [8] did not show any evidence of possible harm elicited by the QoL pathway. In the long-term, this study is also important for improving QoL of individual oncological patients. There are no known risks for participating clinicians or CPs. The study is being conducted in compliance with ethical and scientific standards [37, 38]. We do not anticipate the need for provisions concerning post-trial care. If modifications of the study protocol (protocol version 2.5, September 16, 2024, are made (e.g., changes to eligibility criteria, outcomes, statistical analyses), all investigators will be informed, and changes will be made in ClinicalTrials.gov.

### Primary and secondary outcomes

Concerning primary objective 1, two endpoints will be analysed. The first endpoint is binary: Treatment is defined as successful (responder) if all eight dimensions of the QoL profile reach a value of ≥ 50 points 6 months after baseline (0 = very bad; 100 = very good). If the value of at least one QoL dimension is < 50 points, the treatment is considered unsuccessful (nonresponder), as substantial restrictions in even one QoL dimension can have a negative impact on a patient's overall QoL. As lung cancer is a serious disease with a short median survival time of approximately one year, QoL at 6 months after baseline was chosen as the timepoint to assess the primary endpoint [2, 39, 40]. We expect this period to be long enough to reveal significant effects of the investigated QoL intervention. The second endpoint, enhancing the credibility of the first primary endpoint, are the rates of patients with a QoL score ≥ 50 points separate for each dimension of the QoL profile 6 months after baseline. Both endpoints formulated above will be tested in strict order (1), (2); that is, a statistical testing procedure with a priori ordered hypotheses will be applied [41].

Concerning primary objective 2, QoL data will be supplemented with clinical cancer registry data to answer relevant medical and therapeutic questions. More research questions will be defined in two workshops using a nominal group technique with a group size of 7 to 15 participants (physicians, patients) [42].

Concerning secondary objectives 3–6, a variety of items concerning the perception of the QoL monitoring system will be analysed exploratively.

### Sample size

Trial sample size calculation is based on the first primary endpoint "responder", i.e. the relative frequency of patients with QoL scores in all eight dimensions ≥ 50 after 6 months (i.e. without a need for QoL therapy in the profile). This endpoint was used in RCTs on patients with breast cancer [9] and colorectal cancer [8]. As there is a lack of empirical evidence supporting this endpoint for patients with lung cancer, we are using the RCT protocol for patients with colorectal cancer as a guide. These patients are likely to have QoL deficits comparable to those of patients with lung cancer. We expect that 45% of patients in the intervention group will have no need for QoL therapy 6 months after treatment compared to 25% in the control group. With an α of 5%, 89 patients are needed in each group to detect the hypothesised difference with a targeted power of 80% (1-β) when using the Χ^2^-test of independence. To compensate for approximately 20% drop-outs (death, refusal) within the 6 month observation period, we will randomize 110 patients per group. Thus, the total sample size will be 220.

### Statistical analyses

All variables will be presented using descriptive statistics (frequencies, percentages, means/standard deviations, medians/interquartile ranges, and various graphical representations). Point estimates will be accompanied by confidence intervals.

The design for the endpoints of primary objective 1 is based on a method of fixed a priori ordered hypotheses [41]. Thus, the second primary endpoint will be analysed only confirmatory at an α of 5% if the null hypothesis of the first primary endpoint can be rejected (*p* < 0.05). This design assures a global alpha of 0.05. The analyses of the primary endpoints (including both the first and second primary endpoints) will be conducted using the Χ^2^-test of independence, setting the level of statistical significance at *p* < 0.05.

Additionally, a multivariable regression analysis in the sense of a sensitivity analysis is planned to adjust for potential risk factors. It is not planned to use imputation to handle missing data in the primary analysis. However, missing data will be accounted for in sensitivity analyses using the following methods: imputation of the last available QoL measurement (last observation carried forward (LOCF) approach); definition of patients with missing data as nonresponders. Analyses will be performed in the intention-to-treat population (sensitivity analysis in the per-protocol population). Statistical tests regarding the secondary endpoints will also use the *p* < 0.05 significance threshold, but all secondary results will be interpreted in an explanatory manner. Secondary analyses will include analyses of covariance (ANCOVAs) using the continuous QoL data as the dependent variable and sex, age, study region, and baseline QoL as covariates.

### Data management and privacy

The web application is password-protected and is stored on a web hosting server at the University of Regensburg. At study entry, data of the first QoL measurement are entered into the web application by a study coordinator of the study coordination unit at the Tumor Center Regensburg (TCR) and are controlled by a second, independent member of the study coordination unit. Further QoL measurements can be directly entered by the patients via their mobile phone or computer so that errors at data entry are avoided. A QoL questionnaire can be completed only if all questions are answered so that data completeness is ensured.

A data protection concept was created. All the data will be pseudonymised. The study coordination unit (TCR) maintains pseudonymisation lists, handles communication with patients, administers the electronic survey platform, monitors data entry, and communicates the follow-up survey to the included patients and CPs.

**Fig. 5:**
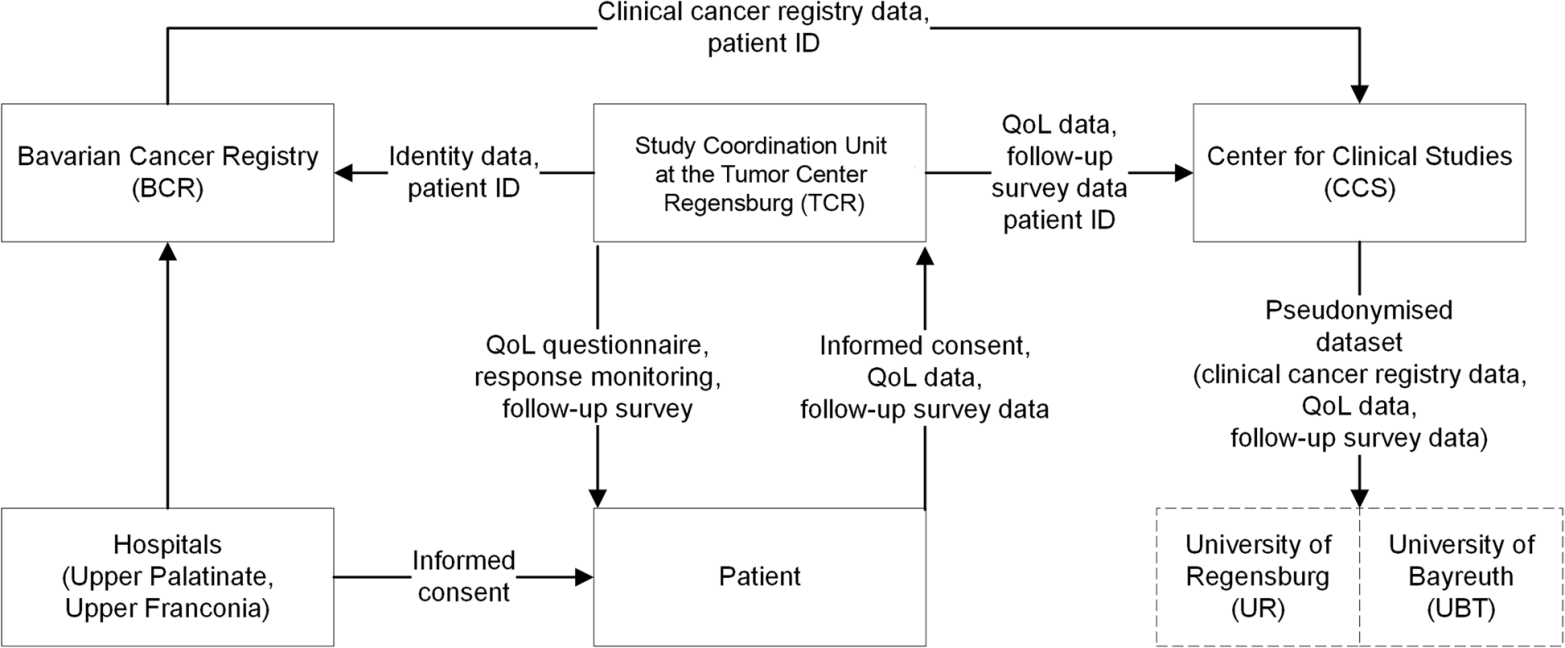
Data flow

Before linking QoL data with data from the Bavarian Cancer Registry (BCR), a statement is requested from the Advisory Board of the BCR. The study coordination unit (TCR) transmits the identity data of study patients with a patient ID to the study coordination unit of the BCR. At the same time, QoL study data are transmitted by the study coordination unit (TCR) with the patient ID to the Center for Clinical Studies (CCS). The BCR also transmits the clinical cancer registry data, provided only with the patient ID, without personally identifiable data, to the CCS, where they are linked to the QoL data using the patient ID. The CCS then makes the final pseudonymised data set available to the University of Bayreuth (UBT) and the University of Regensburg (UR: study research unit of the TCR) (see Fig. 5). To ensure a strict separation between the study coordination unit and the study research unit of UR/TCR, the following measures will be taken: The pseudonymised data set to be evaluated will be stored on a local server at the TCR. The evaluation of this data set is carried out by employees who are specifically responsible for data analysis in a room on the ground floor separate from the study coordination unit. They have no access to the server with the patients' identity data. The database with the identity data is located on a server of the University of Regensburg. Only the employees of the study coordination unit have access to these patient data. The operating systems are protected with a password. The data are accessed via an encrypted TrueCrypt container with a password. This is only opened when data analyses are performed. The data are stored in accordance with applicable standards and retention periods. Anonymized individual QoL data (IPD) that underlie the results in a publication are planned to be made available upon the request of scientists.

## Discussion

This study investigates the effectiveness of a patient- and preference-oriented QoL monitoring system in patients with lung cancer based on comprehensive work involving a complex intervention including two RCTs [8, 9]. In the intervention group the treatment of cancer patients’ QoL will be just as standardised as medical therapy, which is a major strength of this study. To our knowledge, this is the first RCT investigating a QoL intervention that is based on patient and physician preferences. Another strength of this trial is that QoL is not only measured and results are presented to physicians but also that there is a network of local healthcare providers for different regions available to help physicians and their patients receive specific support in case of reduced QoL. In contrast to other interventional trials on QoL, the setting of this study is not limited to the inpatient sector or outpatient care in only one or a few centres but encompasses multiple medical practices, including physicians with different specialisations, so that there is a high degree of external validity. Broad inclusion criteria and the implementation of the intervention in two different German regions additionally increase external validity so that the study is expected to have a high degree of generalisability. Another strength is that the QoL data generated in this study are linked with routine clinical cancer registry data. Through this, an integrated view of medical issues can be taken by including the patient perspective. This helps to generate new knowledge regarding the potential of linking cancer registry data with QoL data for future health services research.

However, there are also some limitations that need to be considered. Because of the complexity of the intervention, it might be difficult to determine which specific facet of the intervention is responsible for an effect. Therefore, the QoL therapies patients receive are recorded and investigated for effectiveness in subgroup analyses. Moreover, it is possible that there are outpatient practices that do not want to participate in the study, reducing generalisability. To ensure high participation rates, clinicians will inform CPs by phone about the willingness of their patients to participate in the RCT and motivate CPs to participate in the study. Moreover, CPs benefit from the participation of their patients in the study, as they have access to the list of healthcare providers and—provided that their patient is part of the intervention group—they can also see the results of QoL measurements.

The study results will be disseminated through full-text publications and presentations at conferences as well as through the transfer of the results to the medical education of physicians and students. In the long term, the patient- and physician-oriented QoL monitoring system is planned to be implemented for the whole region of Germany.

## Trial status

Protocol version 2.5. Recruitment for the trial began on February 15, 2024, with an estimated completion date of August 31, 2025.

## Data Availability

Personnel from the participating institutions directly involved in the study and regulatory institutions will have direct access to trial-related records for monitoring, auditing, and inspection purposes.

## Abbreviations

BCR: Bavarian Cancer Registry
CCS: Centre for Clinical Studies, University Hospital Regensburg
CP: Coordinating practitioner
DCE: Discrete choice experiment
QoL: Quality of life
RCT: Randomised controlled trial
TCR: Tumor Center Regensburg, Center for Quality Management and Health Services Research, University of Regensburg
UBT: University of Bayreuth
